# Toxicological profile of *Amanita virosa* – A narrative review

**DOI:** 10.1016/j.toxrep.2019.01.002

**Published:** 2019-01-09

**Authors:** Milad Tavassoli, Asma Afshari, Andree Letiţia Arsene, Bruno Mégarbane, Josef Dumanov, Monica Maria Bastos Paoliello, Aristidis Tsatsakis, Félix Carvalho, Mahmoud Hashemzaei, Gholamreza Karimi, Ramin Rezaee

**Affiliations:** a Department of Nutrition, Faculty of Medicine, Mashhad University of Medical Sciences, Mashhad, Iran; b Department of General and Pharmaceutical Microbiology, Faculty of Pharmacy, “Carol Davila” University of Medicine and Pharmacy, 6 Traian Vuia Street, 020956, Bucharest, Romania; c Department of Medical and Toxicological Critical Care, Paris-Diderot University, INSERM UMRS-1144, Paris, France; d Mycological Institute USA EU, SubClinical Research Group, Sparta, NJ 07871, United States; e Graduate Program in Public Health, Center of Health Sciences, State University of Londrina – UEL, Londrina, Paraná, Brazil; f Department of Molecular Pharmacology, Albert Einstein College of Medicine, Bronx, NY, 10461, USA; g Center of Toxicology Science & Research, Medical School, University of Crete, Heraklion, Crete, Greece; h UCIBIO, REQUIMTE, Laboratory of Toxicology, Department of Biological Sciences, Faculty of Pharmacy, University of Porto, Rua de Jorge Viterbo Ferreira 228, 4050-313, Porto, Portugal; i Department of Pharmacodynamics and Toxicology, School of Pharmacy, Zabol University of Medical Sciences, Zabol, Iran; j Department of Pharmacodynamics and Toxicology, Faculty of Pharmacy, Mashhad University of Medical Sciences, Mashhad, Iran; k Pharmaceutical Research Center, Institute of Pharmaceutical Technology, Mashhad University of Medical Sciences, Mashhad, Iran; l Clinical Research Unit, Faculty of Medicine, Mashhad University of Medical Sciences, Mashhad, Iran

**Keywords:** Mushroom poisoning, Epidemiology, Phalloidin, Amanitin, *Amanita virosa*

## Abstract

Mushrooms account for a part of human diet due to their exquisite taste and protein content as well as their promising health effects unveiled by scientific research. Toxic and non-toxic mushrooms frequently share considerable morphological similarities, which mislead the collectors/consumers, resulting in mycotoxicity. Numerous mushroom species are considered “poisonous” as they produce dangerous toxins. For instance, members of the genus *Amanita*, especially *A. phalloides*, *A. virosa* and *A. verna*, are responsible for severe and even life-threatening noxious consequences. Globally, mushroom poisoning is a crucial healthcare issue as it leads to a considerable number of deaths annually. However, no definite antidote has been introduced to treat this poisoning. The present article discusses the characteristics of *A. virosa* in terms of epidemiology, mechanisms of toxicity, poisoning features and management.

## Highlights

• Globally, mushroom poisoning leads to a considerable number of deaths annually. However, no definite antidote has been introduced yet.
• A mushroom-poisoning outbreak occurred in 2018 in Iran; this overview presents geographical distribution of *Amanita virosa* along with studies reporting *A. virosa* poisonings.
• Also, main toxins of *A. virosa*, their toxicity mechanisms and pharmacological management of mushroom-poisoned individuals are presented.

## Graphical abstract

**Figure:**
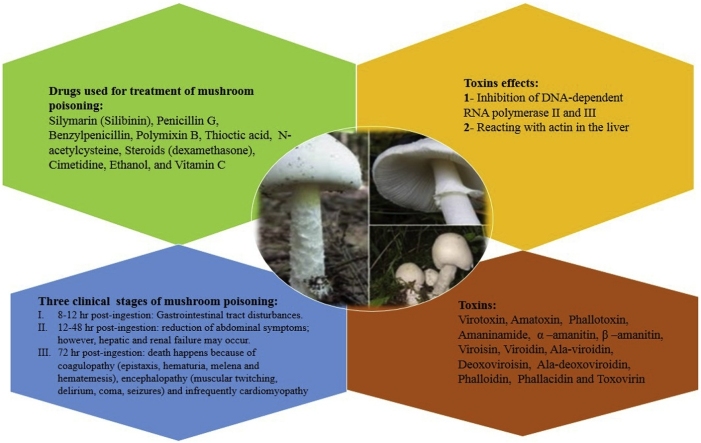

## 1 Introduction

Mushrooms are increasingly found in human diet due to their exquisite taste and protein content as well as their health-promoting effects revealed by numerous scientific studies [[1], [2], [3], [4], [5], [6]]. In this regard, several pharmacologically active compounds have been characterized in mushrooms [7]. Historically, it was noted that athletes in the 3^rd^ century BC consumed mushrooms to enhance their performance [8]. In many countries, including Iran, collection and consumption of wild mushrooms found in forests and grasslands are traditional social activities [4,[9], [10], [11], [12]]. Different types of wild mushrooms are routinely picked and eaten by local inhabitants. Of more than 2000 mushrooms species, about 50 are toxic to humans [13]. Despite marked morphologic similarities, discrimination between toxic and non-toxic mushrooms is usually based on experience-related knowledge and observation. However, increasing interest in wild edible mushrooms has led to frequent collection and ingestion of poisonous species leading to poisonings with persistent issues in diagnosis and management [4,9,14].

Information on mushrooms poisoning in Iran has not been thoroughly recorded. It is therefore hard to retrieve informative data. However, recent reports showed that *A. virosa* is the most-prevalent *Amanita* species in Iran, consistent with reports from East Asian and European countries [15,16]. Additionally, in 2018, an outbreak of mushroom poisoning, later found to be caused by *A. virosa*, took lives of people in Western Iran [17]. Since our preliminary literature search showed no recent comprehensive review on *A. virosa* toxicological profile, we aimed to review the toxic effects of *Amanita* mushrooms, with a special focus on *A. virosa*.

## 2 Epidemiology of mushroom poisonings

Mushrooms poisoning is known as a major problem in Western countries [16,18] representing about 5.8% of the total poisonings in the US [11]. According to a report published by Litovitz et al. in 2002, 8996 mushroom poisoning cases were documented by the American Association of Poison Control Centers (AAPCC). Of these, 576 cases had mild poisoning, 56 had severe clinical conditions and six individuals died [19]. The Annual Report of the National Register of poisoning (U.S., 2009) reported 4083 (73.9%) children with mushroom poisoning with 3012 (54.5%) of them being <6 years old [20]. Later, in 2016, 6421 mushroom-poisoned cases were reported to AAPCC, including 39 severe and two fatal cases [21]. In Turkey, 143 mushroom-poisoned patients were admitted over a four-year period (between 1996 and 2000) to the central hospital of Osmangazi University, of which four patients died [4]. Another report from Turkey, reported 62 deaths in children aging 0–18 years, between 2009 and 2013 in Trabzon. It was found that 4 children (6.5%) were died due to mushroom poisoning; 3 of them were 0–3 years old [22].

In Switzerland, in a retrospective study conducted on 6307 patients with mushroom exposure (from 1995 to 2009), *A. virosa* was regarded as the cause of toxicity in one mild and 1 moderate cases. Generally, it was described that fatal poisonings were caused by amatoxin-containing species [23].

In Iran, *Amanita* species grow in many forested regions such as Mazandaran and Gilan, Northern Iran, as well as Azerbaijan (north-western Iran) and Western provinces [15,20,[22], [23], [24], [25], [26], [27]], but scarce information is available concerning mushrooms poisoning [24,25]. In 1993, three cases of *A. virosa* poisoning were reported from Hamadan, Western Iran, where one patient died [15]. The epidemiological pattern of mushroom poisoning among children aged 11–15 years admitted between 1988 and 1993, to Loghman Hakim Hospital, Tehran, Iran showed a mortality rate of 71% [24,28]. Another report from the same hospital indicated that from eight mushroom-poisoning cases, two patients (25%) died due to hepatic encephalopathy and gastrointestinal bleeding [29]. In a study conducted in 2006 in Iran, 72,421 suspected cases were examined and 37 patients (68% male and 32% female with an average age of 31 years old) were found to be intoxicated by poisonous mushrooms [24]. Another study reported 32 mushroom-poisoned patients (with an average age of 24.6 years old) referred to the Toxicology Center of Mashhad, Khorasan Razavi, Eastern Iran, from 2005 to 2011. Mushroom intoxication represented 0.1% of all intoxication cases admitted to the hospital [26].

In the most recent outbreak of mushroom poisoning in Iran, 1200 intoxicated individuals were referred to hospitals in 13 Western and Northwestern cities of Iran (over 90% of patients were from Kermanshah, Lorestan, Kordestam, and West Azerbaijan provinces) [25]. Of these patients, 8.9% were hospitalized and 1.5% died. Early signs and symptoms included abdominal pain, nausea, vomiting, and diarrhea. Though 50 toxic species of mushrooms grow in Iran, this recent report held *Lepiota brunneioncarnata*, *Hypholoma fascicalare*, and *Coprinopsis atramentaria* responsible for this outbreak [25]. Nevertheless, according to Food and Drug Administration, Health Ministry of Iran (available at https://www.tehrantimes.com/news/423947/Mushroom-poisoning-kills-18-in-Iran), *A. virosa* was the cause of poisoning in this scenario [17].

Variations in the mortality rate can usually be attributed to the type of mushroom species ingested, different levels of included toxins, and the vulnerability of poisoned subjects [26]. In most studies, spring and autumn were shown to have the highest incidence rates of mushroom poisoning [17,23,27,30,31].

## 3 Classification and toxicity of *Amanita* mushrooms

**Fig. 1:**
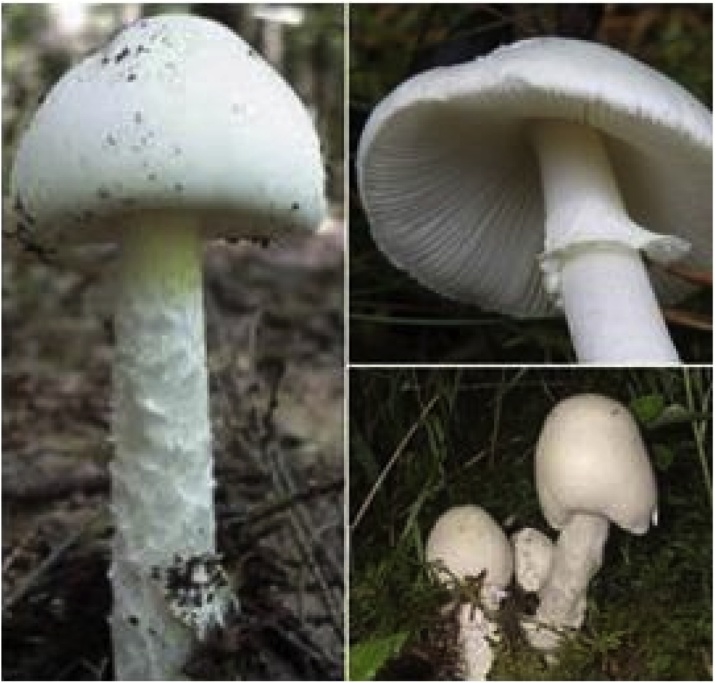
Amanita virosa.

Numerous toxic mushrooms are found around the world, including those containing cyclopeptides, usually regarded as the most toxic species [32]. The genus *Amanita* belongs to the family *Amanitaceae* and includes the majority of mushrooms that are toxic to humans [18]. *Amanita* genus has about 900 to 1000 species, of which nine are known to produce poisonous amatoxins. Although the genus *Lepiota* has the largest number of amatoxin-producing species, the species from the *Amanita* genus are responsible for most of mushroom-poisoning deaths [[32], [33], [34], [35]]. The most well-known species of *Amanita* are *A. phalloides, A. virosa*, and *A. verna*; also, *A. muscaria*, *A. smithiana*, *A. thiersii*, *A. ocreata*, *A. suballiacea*, *A. tenuifolia*, *A. nauseosa, A. virgineoides* and *A. bisporigera* are other members of this genus. Among these different species, *A. phalloides, A. verna*, and *A. virosa* exert the highest toxicity, mainly involving the liver, kidneys and central nervous system [33,36,37]. More than 90% of the mushroom-related fatalities that are attributed to these *Amanita* mushrooms in Central Europe and North America, result from life-threatening acute hepatitis. Three other species, *A. exitialis*, *A. fuliginea* and *A. subjunquillea*, found in East Asia, contain cyclopeptides and have also been shown to cause liver failure and death [10,33,[37], [38], [39], [40], [41], [42], [43]]. The other *Amanita* mushrooms mainly cause nephrotoxicity [[44], [45], [46], [47]]. Interestingly, *A. muscaria* and *A. pantherina* contain ibotenic acid and muscimol, which produce hallucinogenic effects in addition to acute renal failure [37,44,48,49]. Among the rare edible species of *Amanita* mushrooms, *A. lanei* is frequently mistaken by species of the genus *Agaricus* [34]. *A. virosa* has a pure white appearance, like a veil of angels, and its roots are smoother compared to *A. verna*, but due to its deadly nature, it has been called "The destroying angel" (Fig. 1) [18]. Like other *Amanita*'s mushrooms, it has a sweet smell and taste. The color of *A. virosa* cap is white and the color of the center becomes yellow or brown as it matures. *A. virosa* has white spores of 8–10 mm in diameter, with a length-to-width ratio <1.25 (Fig. 1) [18,34]. One of the most beautiful and widespread species of *Amanita* is the red and white *A. muscaria* also known as "fly agaric" [50].

Since a long time, three types of mushrooms namely, *A. virosa*, *Russula vesca* and *Russula persicina*, have been identified in Iran [27]. Recent studies have shown that in Iran, *A. virosa* is more prevalent than *A. phalloides* [15,[24], [25], [26], [27]].

## 4 Toxins of *Amanita* mushrooms and mechanisms of toxicity

Major toxin classes found in the genus *Amanita* are amatoxins, phallotoxins and virotoxins, all classified as cyclopeptides with a sulfur-linked tryptophan and some unusual hydroxylated amino-acids [51]. Amatoxins are at least eight related toxic compounds of eight amino-acid residues arranged in a conserved pentacyclic structure. Phallotoxins are at least seven compounds, all of which are bicyclic heptapeptides. Virotoxins are monocyclic peptides formed by at least five different compounds. Their structure and biological activity are similar to those of phallotoxins, thus suggesting that they share common precursor pathways.

**Fig. 2:**
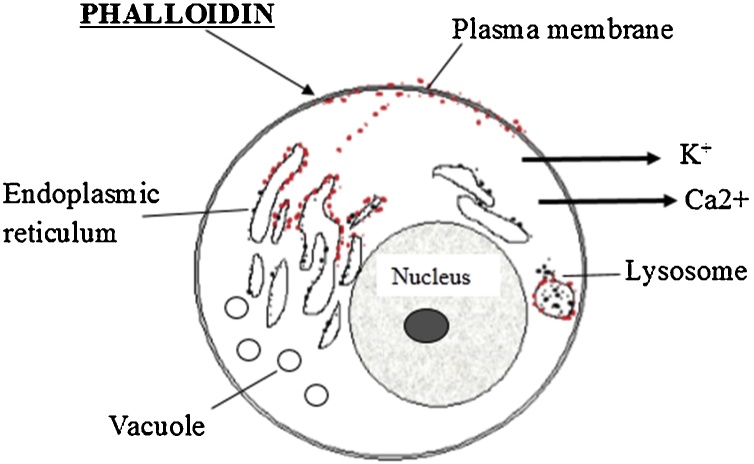
Phalloidin attacks the cell membranes causing leakage of calcium atoms, followed by loss of potassium ions. Reproduced based on a previously published report [60] with permission from the Estate of Bunji Tagawa.

**Fig. 3:**
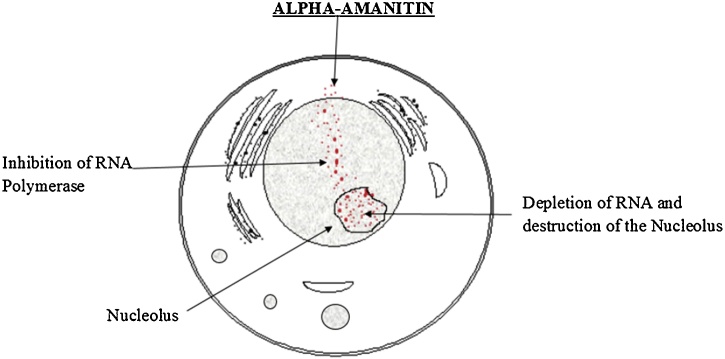
Amanitin disintergrates hepatic cell nucleus. Reproduced based on a previously published report [60] with permission from the Estate of Bunji Tagawa.

The two main toxins of *A. phalloides* are named phalloidin and amanitin. Phalloidin (MW of 900 Da) was first identified by Wieland in 1937 [52] in *A. phalloides* while phallotoxins were found in *A. virosa* for the first time in 1974 [53]. Amanitin, mainly alpha-amanitin with MW of around 900 Da [16], was discovered in *A. virosa* in 1966 [54], although its presence in this species remained controversial [55]. Interestingly, while amaninamide may be found in *A. virosa*, γ-amanitin is produced by *A. phalloides* and other *Amanita* species [37,56]. To date, the majority of the studies considered that *A. virosa* contains two major amatoxins namely alpha-amanitin and beta-amanitin, and two phallotoxins namely phalloidin and phallacidin [18,57,58]. However, in a study performed by Buku in 1980, amaninamide and alpha-amanitin but not beta- amanitin, were found in *A. virosa*. These results were supported by the findings reported by Yocum and Simons, following the chemical characterization of *A. virosa* mushrooms collected from different US regions [55,57,59]. Phalloidin and alpha-amanitin mechanisms of action are presented in Fig. 2, Fig. 3.

**Fig. 4:**
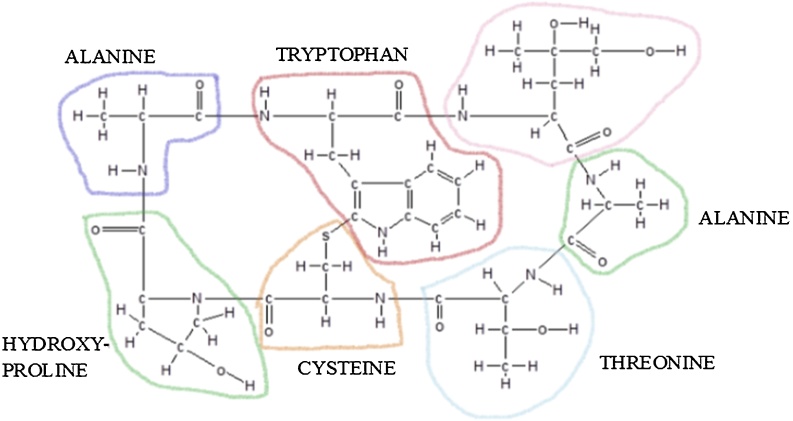
Phalloidin as a phallotoxin, is a cyclic, or ring molecule made up of seven amino acids (outlined). A sulfur atom, connects the side chains of two amino acids on opposite sides of the ring. Reproduced based on a previously published report [60] with permission from the Estate of Bunji Tagawa.

**Fig. 5:**
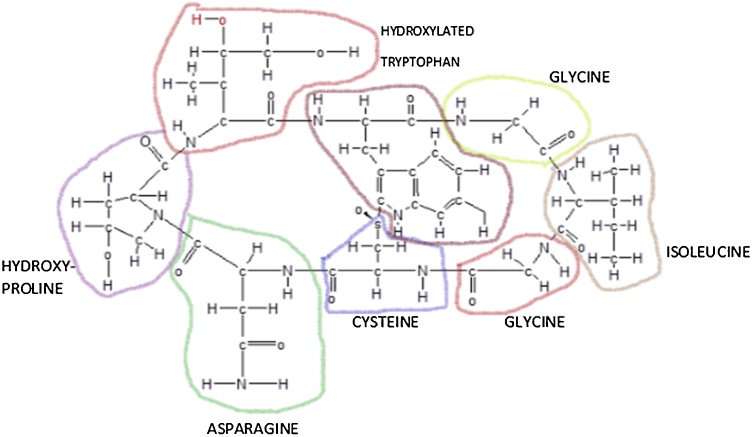
Eight amino acids of amatoxin. Reproduced based on a previously published report [60] with permission from the Estate of Bunji Tagawa.

Virotoxins are monocyclic peptides, and in terms of biological activity they are similar to phallotoxins [[61], [62], [63]]. Some articles indicated that virotoxins are only present in *A. virosa*, but other studies found virotoxins in *A. subpallidorosea* and *A. virosa* species. *A. subpallidorosea* and *A. virosa* are clustered according to phylogenetic analysis. Also, according to previous studies, the characteristics of toxin cyclopeptide are consistent with phylogenetic molecular relationships [56,57,64]. Virotoxins were mainly found in mushrooms collected from Europe and North-America. Toxovirin isolated from *A. virosa* has no mono- and diamino-oxidase activity but high oxidase activity for specific amino acids. Toxovirin-related effect on L-amino acids is double than that for DL-racemic mixtures. This toxin is chemically and structurally similar to toxophallin isolated from *A. phalloides* [65]. Viroidin cyclopeptide, a monocyclic peptide, was first detected in 2016 in *A. virosa* [37]. Like *A. phalloides* and *A. virosa*, other *Amanita* species like *A. bisporigera* and *A. verna* can also produce toxic peptides (amatoxin, phallotoxins, and virotoxin) [55,66,67]. Amatoxins have eight amino acids instead of seven. Again, sulfur atom joins two side chains; hydroxyl group (outlined) is essential for toxicity. Alpha-amanitin phalloidin structures (Fig. 4, Fig. 5) were worked out by Theodor Hermann Felix Wieland (1913–1995) at The Max Plack Institute for Medical Research in Heidleberg in 1974 [68].

**Fig. 6:**
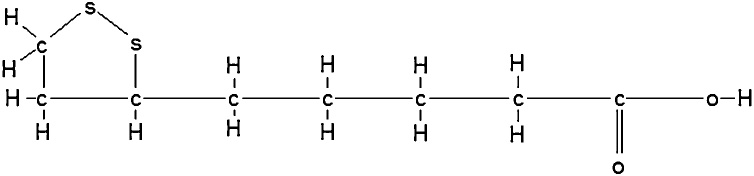
Thiotic Acid chemical structure. Reproduced based on a previously published report [60] with permission from the Estate of Bunji Tagawa.

Thoitic acid (also known as alpha lipoic acid) is a possible antidote to amanita poisons. It contains a ring of carbons and sulfur atoms and a chain of carbon atoms (Fig. 6) [60].

Though phallotoxins and virotoxins act faster, amatoxins are 10–20 times more toxic and are responsible for observed fatalities [37,50]. Amatoxins are potent and selective inhibitors of RNA polymerase II, a vital enzyme in the synthesis of mRNA, microRNA, and small nuclear RNA, leading to protein synthesis interruption and cell death. The lethal dose of orally-administered alpha-amanitin in humans is 0.1 mg/kg [26,69]. Amanitins including those produced by *A. virosa,* can damage the liver, kidneys and brain, eventually causing mortality [65,70,71]. Phallotoxins, although highly toxic to the liver and muscular cells, strongly bind actin [57], but mildly contribute to amanita-related toxicity since they are not absorbed by the gastrointestinal tract. Like phallotoxins, virotoxins have limited toxic effects after oral exposure. Their interaction with actin, which is even weaker than that of phallotoxins, stabilizes the bonds between actin monomers and prevents microfilaments depolymerization. *A. phalloides* and *A. virosa* also produce toxic lectins, differing in their carbohydrate moieties but leading to similar hemolysis [65,72]. Additionally, two amino-acids namely, 2,3-trans-3,4-dihydroxy-l-proline and 20-(methylsulphonyl)-l-tryptophan, were identified in *A. virosa*.

The physicochemical properties of the majority of these toxins make them heat-resistant and food processing methods like grilling, boiling, frying, and steaming cannot completely eliminate them [4,73,74]. Ingestion of toxin-containing *Amanita* mushrooms leads to challenging hepatotoxicity although additional disturbances like allergic gastroenteritis may also result [14,24,52,[75], [76], [77]]. Interestingly, nephrotoxic effects were also demonstrated in different studies [33,[78], [79], [80], [81]], which may have an important impact on the renal excretion of absorbed toxins. Therefore, the final observed clinical toxicity is related to the type and amount of ingested mushrooms, i.e. the content and specific toxicity of the involved cyclopeptides [82]. Different toxins found in *A. virosa* along with their mode(s) of action are presented in Table 1.

**Table 1:** Features of *Amanita virosa* toxins.

Type of toxin	Chemical structure	Target organ	Mechanism of action	Similar toxins	References
Virotoxin	Monocyclic heptapeptides (containing D-serine)	Liver and kidney	Disturbing Ca^2+^ homeostasis and reacting with actin	Ala-viroidin, Viroisin, Deoxoviroisin, Viroidin, Ala-desoxoviroidin and Phallotoxin	[57,64]
Amatoxin	Cyclic heptapeptides	Liver and kidney	Inhibition of DNA-dependent RNA polymerase II	α –amanitin, β -amanitin	[64]
Phallotoxin	Cyclic heptapeptides	Liver and kidney	Reacting with actin in the liver	Virotoxin	[64]
Amaninamide	Bicyclic peptides and (analogous to α –amanitin)	Liver	Inhibition of RNA polymerase II	Amatoxins	[59]
α -amanitin	Bicyclic peptides	Liver	Inhibition of RNA polymerase II	Amatoxin	[34]
β -amanitin	Bicyclic peptides	Liver	Inhibition of RNA polymerase III	Amatoxin	[34]
Viroisin	Cyclopeptide	liver	Reacting with actin in the liver	Virotoxin	[75]
Viroidin	Cyclopeptide	Liver	Reacting with actin in the liver	Virotoxin	[75]
Ala-viroidin	Cyclopeptide	Liver	Reacting with actin in the liver	Virotoxin	[75]
Deoxoviroisin	Cyclopeptide	Liver	Reacting with actin in the liver	Virotoxin	[75]
Ala-deoxoviroidin	Cyclopeptide	Liver	Reacting with actin in the liver	Virotoxin	[75]
Phalloidin	Cyclopeptide	Liver	Reacting with actin in the liver	Phallotoxin	[58,64]
Phallacidin	Cyclopeptide	Liver	Inhibition of RNA polymerase	Phallotoxin	[57,64]
Toxovirin	Cyclopeptide	Liver	Highly toxic against mammalian cells (its L-amino acid oxidase activity induces apoptosis in cancerous cells)	Toxophallin and lectin	[65]

From a toxicokinetic point of view, the liver can excrete about 60% of the toxins in the bile [83] but returns to the liver through enterohepatic recirculation. Alpha-amanitin is quickly cleared from the serum by the kidneys [84]. A large amount of amatoxin is taken by the hepatocytes and undergoes extensive enterohepatic circulation [52,72]. The duration of action of these toxins is about 10–15 h in humans [50].

## 5 Toxic features and management

Most of *Amanita* poisonings are related to *A. phalloides*, *A. virosa* and *A. verna*, respectively. Nonetheless, since the incidence of *A. virosa* intoxication is increasing, more studies should be performed on this species [85].

Most mushroom intoxications are initially presented with gastrointestinal symptoms alone and usually resolve over time, mimicking viral gastroenteritis, but potentially lethal liver dysfunction may occur with *Amanita*. In *A. virosa*-poisoned patients, nausea and vomiting are the most common symptoms [17,23,26,27,30,31]; abdominal pain, diarrhea, irritability, vertigo and hepatitis may also occur. *A. virosa* poisoning develops in three clinical stages, starting 8–12, 12–48 and 72 h after the ingestion, respectively. The pancreas, testicles and blood are also affected by this intoxication. During the first stage the gastrointestinal tract is stimulated this effect is generally attributed to the phalloidin toxin and its active metabolites. The second stage of *Amanita* poisoning presents marked reduction of abdominal symptoms; however, hepatic and renal failure may occur. During the third phase, death happens because of coagulopathy (epistaxis, hematuria, melena and hematemesis), encephalopathy (muscular twitching, delirium, coma, seizures) and infrequently cardiomyopathy [86].

High Performance Liquid Chromatography (HPLC) has been the most common method used for the quantitative and qualitative analysis of *Amanita* mushroom toxins in biological specimens [[87], [88], [89], [90]]. Nevertheless, inconsistencies in methods of extraction of toxins and HPLC conditions do not allow drawing conclusions based on information reported by different laboratories. For instance, deadly cyclopeptidic toxins of *A. fuligineoides* and *A. rimosa* are yet to be discovered [37].

Mushrooms with incubation period <6 h contain muscarine, coprin, ibotenic acid and psilocybin toxins, cause mild clinical symptoms that disappear in a short time [26]. By contrast, this time delay was found to be a major and independent predictor of fatality in amatoxin poisoning [23,26,91,92].

Management of *Amanita* mushroom poisoning is mainly supportive in combination to gastrointestinal decontamination. Activated charcoal efficacy and/or gastric lavage is most useful if attempted within 1 h after the ingestion of a potentially life threatening poison. Of note, activated charcoal (20–40 g every 3–4 h) has also been administered routinely because it may also interrupt the enterohepatic circulation of amatoxins and potentially reduce their toxicity. On the other hand, gastric lavage is contraindicated in patients with loss of airway protective reflexes [93]. The main current goals of mushroom poisoning treatment are to reduce the serum concentrations of mushroom toxins in order to limit the extent of exposure and lessen the risks of organ damage [36,94,95]. Intravenous fluids should be given for forced diuresis and also to replenish fluids and electrolytes lost during the gastrointestinal phase [93]. The use of different extracorporeal techniques to enhance the toxin elimination was successfully reported including plasmapheresis, hemoperfusion, Molecular Absorbent Regenerating System (MARS^®^) dialysis and the fractionated plasma separation and adsorption system (Prometheus^®^). In the presence of acute liver failure caused by *Amanita* poisoning, indication of urgent liver transplantation should be considered, based on the standard King's College Criteria [96].

No specific life-saving approach exists. Various pharmacotherapies have been tested including intravenous penicillin G, thioctic acid, N-acetylcysteine, cimetidine, steroids, polymixin B, vitamin C, silymarin, and silibinin (Table 2) [32,34,36,84,[97], [98], [99], [100]]. Thioctic acid, an antioxidant used in cosmetics and anti-aging products due to its ability to scavenge free-radicals, was successfully used to treat amanitin poisoning cases [36], but has been abandoned, being considered inefficient. Silymarin and N-acetylcysteine have been found to play comparable protective properties mediated through the restoration of hepatic glutathione levels [98]. Recently, polymixin B which has a chemical structure similar to that of alpha-amanitin, has been found effective in preventing mushroom-induced hepatorenal damage [99]. In Iran, treatments such as penicillin G and silymarin, are routinely administered to most patients [100].

**Table 2:** Characteristics of the different compounds used to treat Amanita virosa poisoning.

Compound	Mode of action	Activity against	Dose	References
Silymarin (Silibinin)	Maintenance of hepatic glutathione level (reduces amatoxin uptake in the liver)	*A. phalloides* poisoning	25-50 mg/kg/day	[26,32,34,96]
Penicillin G	Binding plasma proteins, prevention of toxin absorption in the liver and excretion of toxins through the kidneys	Amanitin	1 million units/kg/day	[32,34]
Benzylpenicillin	Inhibition of transporter protein	alpha-amanitin	NS	[32,34]
Polymixin B	Binding RNA polymerase II	alpha-amanitin	NS	[34]
Thioctic acid	Acting as a coenzyme in the oxidative decarboxylation of pyruvate	Amanitin	300 to 600 mg/kg with glucose	[53]
N-acetylcysteine	Hepatoprotective activity in acetaminophen poisoning but not in mushroom poisoning	NS	NS	[32,34]
Steroids (dexamethasone)	Controversial reprots	NS	20–40 mg intravenously	[101]
Cimetidine	Suppression of amatoxins metabolism to toxic metabolites	Amatoxins	NS	[34,95]
Ethanol	Induction of toxin uptake by liver cells	Toxins	Solutions of 30–33%	[53]
Vitamin C	Antioxidant activity	Toxins	3 g/day	[53]

NS: not specified.

Toxic mushrooms cannot be easily identified based on their appearance. Mushroom-induced morbidities may result from consumer negligence and the delay for medical consultation after the ingestion of suspected mushrooms. Increasing public awareness seems an appropriate and effective approach to prevent mushroom poisoning. Education is the most effective way for preventing the toxicity. For training and educating the health professionals and public, full instructions provided by the International Chemical Safety Program (IPCS) team can be used [24].

## 6 Conclusions

Recent studies suggest that *A. virosa* is the most prevalent cause of mushroom poisoning in Iran, similar to some Asian and Eastern European countries. Like the well-known *A. phalloides, A. virosa* may be responsible for life-threatening toxic liver failure and even death. These two Amanita species share similar toxins, including amatoxins, phallotoxins and virotoxins. Poisoning management is supportive, although various specific therapies have been used with currently low-level evidence of usefulness to reduce the risks of morbidities and death. Prevention is thus essential and mainly based on information and education.

## Conflicts of interest

None.
